# Cannabidiol in Adults With Lennox–Gastaut Syndrome: Real‐World Experience

**DOI:** 10.1111/ene.70760

**Published:** 2026-09-26

**Authors:** Pyae Aung, Debbie Miller, Emily Sewell, Laura Mantoan Ritter, Evangelia Theochari, Ioannis Stavropoulos, Robert Delamont, Mark P. Richardson, Joel S. Winston, Lina Nashef

**Affiliations:** ^1^ Department of Neurology King's College Hospital London UK; ^2^ Department of Basic and Clinical Neuroscience Institute of Psychiatry, Psychology and Neuroscience, King's College London London UK; ^3^ Department of Clinical Neurophysiology King's College Hospital London UK

**Keywords:** cannabidiol, epilepsy, Lennox–Gastaut syndrome, real‐world evidence, seizure control

## Abstract

**Background:**

We report a single centre experience of cannabidiol use as adjunctive treatment in adults with Lennox–Gastaut Syndrome (LGS).

**Methods:**

Retrospective review of adults with LGS treated with cannabidiol with clinical data collected from electronic records and caregiver standardised seizure diaries with seizure‐related outcomes assessed at baseline, 6 months, and last follow‐up.

**Results:**

Seventy‐nine adults (median age 29 years; 40 male) were included. Median follow‐up was 41 months. Four patients died unrelated to cannabidiol treatment. Sixty‐seven (86%) remained on treatment at last follow‐up. Across all seizure types, 59.5% achieved ≥ 50% reduction at 6 months and 67.1% at last follow‐up. Among those with ‘Drop Seizures’ (*n* = 78), 50% achieved > 50% reduction at 6 months and 61.5% at last follow‐up. ‘Drop Seizure’ responder rates were numerically higher with clobazam > 5 mg/day, but with no statistically significant dose–response relationship seen. Seizure‐free days increased (baseline 5.7/month; last follow‐up 12.5/month). Seizure‐related hospital admissions and injuries declined. Cognitive or behavioural improvements were reported in 48 patients (60.8%); there was no statistically significant association with achieving ≥ 50% seizure reduction. Drowsiness (*n* = 33) was the most frequent adverse event often related to drug–drug interactions. Diarrhoea (*n* = 24) was more common than previously reported. Side‐effects necessitated ASM adjustments in 51 patients. Fourteen were able to withdraw one or more ASMs.

**Conclusions:**

CBD was associated with sustained effectiveness and good tolerability in adults with LGS, with high retention and improvement in seizure and other clinically meaningful outcomes. Adverse effects, mainly interaction‐related, were usually managed with concomitant medication adjustments.

## Graphical abstract

Cannabidiol showed sustained effectiveness and good tolerability in adults with LGS, with high retention over a median follow‐up of 41 months. Treatment was associated with improvement in seizure burden, increased seizure‐free days, fewer seizure‐related hospital admissions, and caregiver‐reported improvement in cognitive and behavioural functioning. Adverse effects, mainly interaction‐related, were usually managed with concomitant medication adjustments.

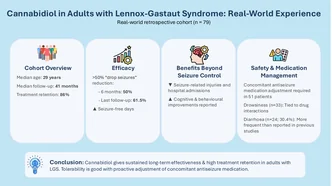

## 1 Introduction

Lennox–Gastaut Syndrome (LGS) is a Developmental Epileptic Encephalopathy (DEE) characterised by (1) multiple seizure types (at least one of which must include tonic) with onset prior to 18 years; (2) cognitive and often behavioural impairments, which may not be apparent at seizure onset; and (3) characteristic electroencephalographic (EEG) features, including slow background, diffuse slow spike‐and‐wave discharges and generalised paroxysmal fast activity [1, 2]. The presentation can be caused by diverse aetiologies [2]. The majority of patients present under 8 years of age, and onset during the second decade is uncommon [3, 4]. Both seizure types and EEG features may evolve over time and may not persist into adulthood [5, 6, 7, 8]. The estimated number of individuals affected by LGS ranges from 1.9 to 60.8 per 100,000—a wide range that may partially reflect variation in threshold or criteria for diagnosis [9]. Seizures in LGS are typically resistant to treatment. Despite therapeutic intervention, most patients continue to experience disabling seizures, and seizure freedom is rarely achieved [10, 11].

Cannabidiol (CBD), a non‐psychoactive component in *Cannabis sativa* [12], has emerged as a promising adjunctive therapy for LGS. Clinical trials have demonstrated its efficacy in reducing seizure frequency [13, 14, 15, 16]. The exact mechanism of action underlying the reduction in seizures is not fully understood. It is known to interact with multiple molecular targets involved in neuronal excitability. Proposed key actions include antagonism of G protein‐coupled receptor‐55 (GPR55) and desensitisation of Transient Receptor Potential Vanilloid‐1 (TRPV1) channels, both reducing intracellular calcium. Another proposed mechanism is inhibition of the equilibrative nucleoside transporter 1 (ENT‐1), which increases extracellular adenosine concentration, which contributes to seizure control [17].

Cannabidiol (CBD; Epidyolex, 100 mg/mL oral solution) was first approved by the U.S. Food and Drug Administration (FDA) in 2018 and subsequently received European Medicines Agency (EMA) authorisation in 2019 as adjunctive therapy for seizures associated with LGS and Dravet syndrome and in 2021 for tuberous sclerosis [18, 19] The National Institute for Health and Care Excellence (NICE) in the United Kingdom approved cannabidiol (Epidyolex) with clobazam as a treatment option for seizures associated with LGS in people aged 2 years and older [13]. Real‐world data, particularly in the adult LGS population, remain limited [16, 20, 21, 22, 23]. We report our real‐world experience with cannabidiol therapy in adults with LGS, focusing on seizure outcomes, treatment tolerability, adverse effects, medication interactions and other patient‐reported changes.

## 2 Methods

This was a retrospective audit of clinical outcomes assessing real‐world use of cannabidiol in adults with LGS between January 2019 and April 2025. The outcomes were reviewed based on documentation from routine follow‐up after initiation of treatment compared to baseline. The project operated under UK Health Research Authority (HRA) London South East Research Ethics Committee approval (reference 18/LO/2048; renewed 24/LO/0057) granted to the King's Electronic Records Research Interface (KERRI). This was approved by the KERRI committee at King's College Hospital (KCH).

### 2.1 Cohort

All patients being treated with cannabidiol (Epidyolex) for LGS identified through adult epilepsy clinics within our service during the period of the audit were included. Patients had a diagnosis of LGS based on age of onset, multiple seizure types including tonic and compatible EEG or video‐EEG findings. Eligibility for cannabidiol was in accordance with NICE guidance and ongoing uncontrolled epilepsy despite standard treatment. NICE requires that cannabidiol is prescribed in conjunction with clobazam.

### 2.2 Data Collection

Clinic letters and historical medical records were reviewed for a detailed account of the patient's history, investigations and treatment. Data were collected retrospectively from electronic medical records and caregiver‐maintained seizure diaries using a standardised proforma at baseline and at 6 months post initiation in view of the requirement by NICE to demonstrate sufficient improvement for continuing prescribing. Outcome at last follow‐up was based on clinical notes/letters. Extracted information included patient demographics, epilepsy and seizure history, aetiology of LGS, intellectual disability, comorbidities and previous and current anti‐seizure medications or epilepsy‐specific interventions. Findings from previous EEG and/or video‐EEG recordings were also compiled for future analysis.

Baseline seizure types and average monthly frequencies were recorded based on care‐giver seizure diaries and clinical assessments over a minimum four‐week baseline period prior to cannabidiol initiation, at 6 months after treatment initiation and at last follow‐up. Seizure types were based on caregiver reports and included generalised tonic–clonic seizures (GTCS), tonic seizures, atonic seizures, drop attacks, myoclonic seizures and absences. Outcomes were analysed by seizure type as well as in relation to the category of ‘Drop Seizures’, defined as epileptic events (tonic, atonic, or tonic–clonic seizures) involving the entire body, trunk, or head, that result in a sudden loss of muscle tone or stiffening, potentially resulting in a fall, injury, or slumping in a chair. This definition, used by Devinsky et al. [15], was adopted by NICE to assess sufficient efficacy for continued prescribing, specifically requiring a minimum of 30% reduction in such ‘Drop Seizures’ by 6 months [13, 15].

Additional parameters documented in the standardised proforma included the number of seizure‐free days, use of rescue treatments, hospital admissions related to seizures or seizure‐related injuries and any changes in sleep, behaviour, or cognition. Adverse events, laboratory results (including liver function tests and full blood counts), and changes to concomitant medications were also extracted.

### 2.3 Treatment Initiation and Titration

Treatment was initiated as part of routine clinical care following NICE guidelines with adjuvant clobazam started, if not already part of the treatment regimen, before cannabidiol initiation. Cannabidiol dose was titrated based on clinical response and tolerability. Routine laboratory monitoring included liver function tests, full blood count and renal function tests. Serum concentrations of concomitant antiseizure medications (e.g., phenytoin, clobazam and everolimus) were measured when clinically indicated to monitor for potential drug–drug interactions. Starting doses were equal to or less than those specified in the summary of product characteristics.

### 2.4 Outcome Measures and Follow‐Up

The main clinical outcome was the change in seizure frequency following initiation of cannabidiol, evaluated at 6 months and last follow‐up. The standardised proforma at baseline and 6 months was used for data on seizure type and frequency, changes in seizure‐free days, use of rescue medications, hospital admissions and attendances, cognitive and behavioural changes and sleep pattern alterations. Additional outcomes were based on caregiver observations and routine clinical documentation. Last follow up was either at last available review during the audit period or until another add‐on medication was initiated. This approach was to minimise confounding from subsequent treatment changes and to allow seizure outcomes to be more reliably attributed to cannabidiol.

Adjustments in anti‐seizure medications (ASMs), cannabidiol dosing, adverse events and treatment retention were noted over the whole period. Retention on cannabidiol was evaluated at 3 months, 6 months and last follow‐up. Deaths which were unrelated to cannabidiol treatment were documented but excluded from retention rate analysis.

### 2.5 Statistical Analysis

Descriptive statistical analysis was performed using Microsoft Excel. Continuous variables were reported as median with interquartile range (IQR) or range or mean ± standard deviation (SD), as appropriate. Categorical variables were summarised as frequencies and percentages. Analyses included comparisons of responder rates according to clobazam dose, concomitant valproate use, active vagus nerve stimulation (VNS), and aetiology, as well as the association between seizure response and caregiver‐reported cognitive improvement, using chi‐squared test or Fisher's exact test, as appropriate. Paired comparisons of seizure‐free days between baseline and follow‐up were performed using paired *t*‐tests. A *p* value < 0.05 was considered statistically significant.

## 3 Results

### 3.1 Patient Demographics

A total of 79 patients with LGS were treated during this audit period. Baseline demographic and clinical characteristics are summarised in Table 1. The median duration of follow‐up was 41 months, and the mean duration was 33.5 months (range 6–75 months). Median age was 29 years with an approximately equal gender distribution (40 males, 39 females). Sixty‐six patients (83.5%) had epilepsy onset before the age of 8 years; 11 patients had onset between ages 8 and 13 and one at the age of 16. In one patient, the exact age of onset in childhood was not available. All patients had a history of tonic seizures, multiple seizure types, and compatible EEG changes. Ninety‐one percent had moderate‐to‐severe intellectual disability.

**TABLE 1:** Patient demographics and baseline characteristics.

Total, *n*	79
Gender, *n*	Male: 40; Female: 39
Age	Median:29 [Range: 18–62]
Duration of epilepsy in years, Median (IQR)	26 (13.3)
Aetiology of epilepsy, *n* (%)	
Brain malformations	10 (12.7)
Acquired/perinatal brain damage	11 (13.9)
Known genetic cause	21 (26.6)
Unknown	37 (46.8)
Intellectual disability documented as moderate or severe, (%)	72 (91.1)
Prior brain surgery (see text), *n* (%)	7 (8.9)
VNS–active, *n* (%)	47 (59.5)
VNS switched off, *n* (%)	13 (16.5)
Ketogenic diet, *n* (%)	Previous: 25 (31.7) Current: 1
Regular clobazam prior to cannabidiol, *n*	41
New clobazam prescriptions to allow for cannabidiol initiation as per NICE guidance, *n*	37

Underlying aetiology included acquired/perinatal brain injury (11/79), brain malformations (10/79), confirmed genetic diagnoses (21/79) and unknown (37/79). Brain malformations were heterogeneous and included malformations of cortical development (pachygyria, cortical dysplasia, polymicrogyria, periventricular heterotopia and agenesis of the corpus callosum) and hypothalamic hamartoma. Among patients with acquired/perinatal brain injury, neuroimaging showed chronic changes consistent with hypoxic‐ischaemic injury, including periventricular leukomalacia, ulegyria, encephalomalacia, white matter and basal ganglia injury, parasagittal gliosis, cerebral volume loss and porencephalic lesions with cerebral hemiatrophy. Among the 21 patients with a confirmed genetic diagnosis, pathogenic variants involved genes associated with DEEs including *STXBP1, CDKL5, HNRNPU, PURA, PPP2R5D, WDR62, NEXMIF, CHD8, NAA10, SCN1B* and *ANKRD11*, together with tuberous sclerosis complex, GLUT1 deficiency syndrome and several chromosomal disorders (Table S2).

Forty‐seven patients (59%) had an active VNS and a further 13 an implanted, but inactive, VNS. One patient was on a ketogenic diet with 25 (32%) having previously been treated with this. At baseline, 65 patients (82%) were taking three or more ASMs, with a median of 4 ASMs (IQR: 1; range 1–6). The median number of prior ASMs (excluding current medications) was 6 (IQR: 6; range 1–18). Seven patients (8.9%) had previously undergone epilepsy surgery or tumour resection: three had corpus callosotomy; one a callosotomy then an anterior frontal resection for cortical dysplasia; one surgery for hypothalamic hamartoma; and two with tuberous sclerosis had resective surgery (one left occipital tuber and one subependymal giant cell astrocytoma).

### 3.2 Cannabidiol Treatment

The median age at cannabidiol initiation was 27.1 years (range: 12.9–59.4). Eight patients had initiated cannabidiol therapy as older teenagers under paediatric care (aged less than 18) but had transitioned to adult services. Cannabidiol was offered in accordance with NICE guidance with adjuvant clobazam. Where clobazam was not already part of the regimen, it was generally introduced at least 2 weeks prior to cannabidiol initiation.

At treatment initiation, the median starting dose of cannabidiol was 2.4 mg/kg twice daily (IQR: 0.5). The initiation dose range was 25–260 mg twice daily. Dosing was individualised by the treating clinician, with most patients receiving between 2.0 and 2.5 mg/kg twice daily. In a few patients, if generally intolerant of medication or there was specific concern about potential drug–drug interactions, smaller doses were used. At 6 months, the median dose was 5.1 mg/kg twice daily (IQR: 3.5). The dose range was 25–1000 mg twice daily. At last follow‐up, the median dose was 7.4 mg/kg twice daily (IQR: 4.5; range: 150–1100 mg twice daily). An analysis comparing patients receiving < 5 mg/kg twice daily with those receiving ≥ 5 mg/kg twice daily at last follow‐up showed numerically higher responder rates (> 50% seizure reduction) in the ≥ 5 mg/kg twice daily group (81.1% vs. 71.4%). However, this difference was not statistically significant (Fisher's exact test, *p* = 0.468).

### 3.3 Outcomes

Four (5.1%) and eight patients (10.1%) had discontinued cannabidiol by 6 months and last follow‐up, respectively. Four patients (5.1%) had died from causes unrelated to treatment with cannabidiol after the initial 6‐month period.

#### 3.3.1 Seizure Frequency

**FIGURE 1:**
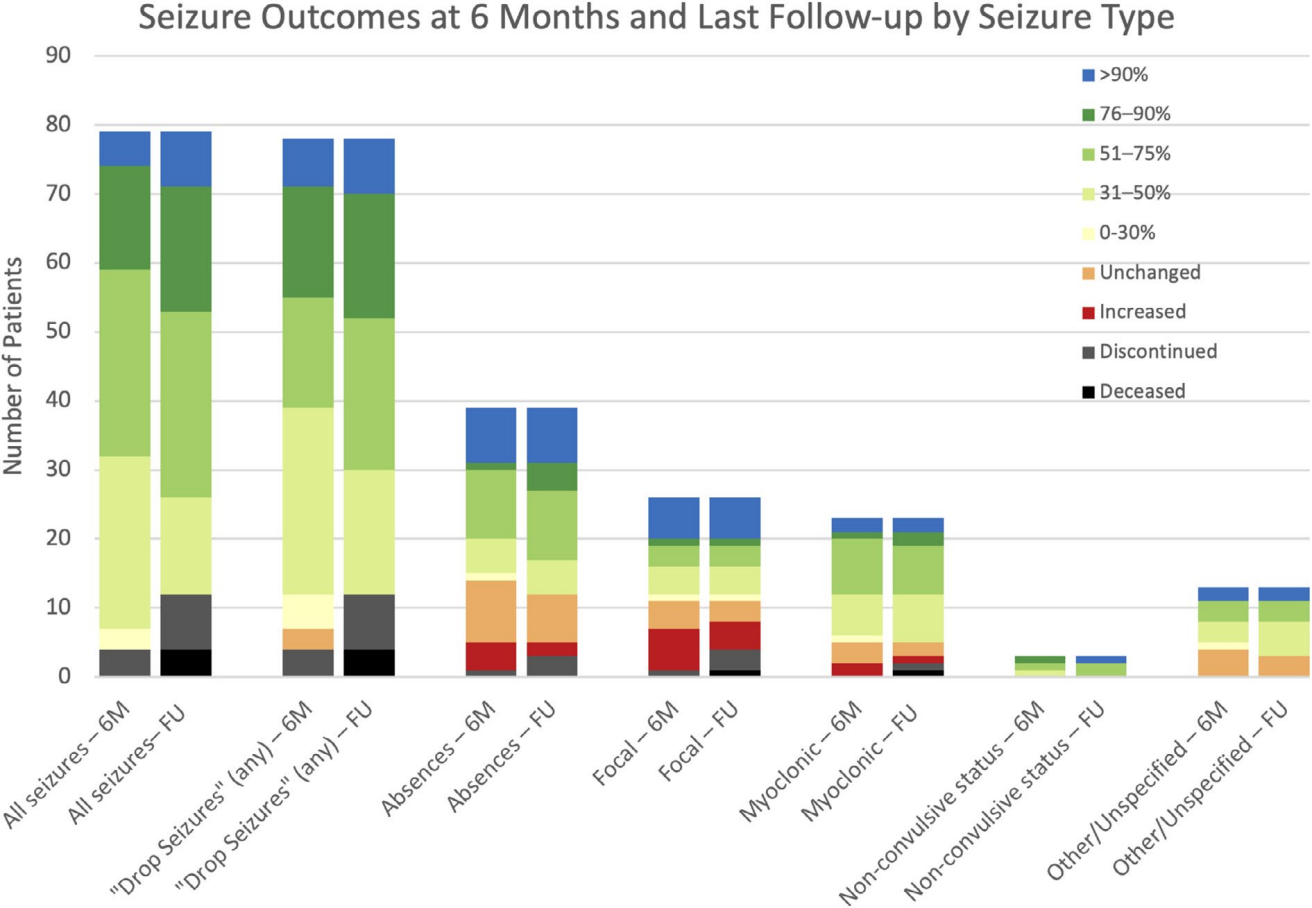
Seizure reduction outcomes following cannabidiol treatment: 6‐month (6 M) and last follow‐up (FU).

Seizure outcomes were assessed, relative to baseline, at 6 months and last follow‐up. A full breakdown of seizure outcomes by type is provided in Tables 2, 3 and Figure 1.

**TABLE 2:** Seizure outcomes at 6 months by seizure type.

	Baseline, *n*	6 M, *n*	Increased@ 6 M, *n* (%)	Unchanged@ 6 M, *n* (%)	Improved seizure frequency					Discontinued@ 6 M, *n* (%)
					0%–30%@ 6 M, *n* (%)	31%–50%@ 6 M, *n* (%)	51%–75%@ 6 M, *n* (%)	76%–90%@ 6 M, *n* (%)	> 90%@6 M, *n* (%)	
‘Drop Seizures’ (any)	78	74	0 (0.0)	3 (3.8)	5 (6.4)	27 (34.6)	16 (20.5)	16 (20.5)	7 (9.0)	4 (5.1)
Tonic	57	57	4 (7.0)	7 (12.3)	3 (5.3)	15 (26.3)	10 (17.5)	12 (21.1)	6 (10.5)	0 (0.0)
Atonic	11	11	0 (0.0)	2 (18.2)	0 (0.0)	2 (18.2)	4 (36.4)	2 (18.2)	1 (9.1)	0 (0.0)
GTCS	69	65	8 (11.6)	7 (10.1)	4 (5.8)	16 (23.2)	16 (23.2)	8 (11.6)	6 (8.7)	4 (5.8)
Head Drops	4	4	0 (0.0)	0 (0.0)	0 (0.0)	2 (50.0)	1 (25.0)	1 (25.0)	0 (0.0)	0 (0.0)
Drop attacks (as labelled by caregivers)	12	11	2 (16.7)	2 (16.7)	1 (8.3)	1 (8.3)	2 (16.7)	3 (25.0)	0 (0.0)	1 (8.3)
Absences	39	38	4 (10.3)	9 (23.1)	1 (2.6)	5 (12.8)	10 (25.6)	1 (2.6)	8 (20.5)	1 (2.6)
Myoclonic Seizures	23	23	2 (8.7)	3 (13.0)	1 (4.3)	6 (26.1)	8 (34.8)	1 (4.3)	2 (8.7)	0 (0.0)
Focal Seizures (with or without impaired awareness)	26	25	6 (23.1)	4 (15.4)	1 (3.8)	4 (15.4)	3 (11.5)	1 (3.8)	6 (23.1)	1 (3.8)
Non‐convulsive status	3	3	0 (0.0)	0 (0.0)	0 (0.0)	1 (33.3)	1 (33.3)	1 (33.3)	0 (0.0)	0 (0.0)
Other/unspecified	13	13	0 (0.0)	4 (30.8)	1 (7.7)	3 (23.1)	3 (23.1)	0 (0.0)	2 (15.4)	0 (0.0)
All Seizure types	79	75	0 (0.0)	0 (0.0)	3 (3.8)	25 (31.6)	27 (34.2)	15 (19.0)	5 (6.3)	4 (5.1)

*Note:* Seizure type classification was based on caregiver seizure charts. Throughout the table, *n* refers to the number of patients. Percentages at 6 months are calculated using the baseline number of patients for each seizure type. ‘6 M, *n*’ includes only patients with available 6‐month data; discontinued patients are excluded from this column but retained in the baseline denominator for percentage calculations. ‘Drop Seizures’ (any) includes patients with one or more of the following seizure types: tonic, atonic, tonic–clonic, head drops, or drop attacks (as labelled by caregivers), based on seizure diaries. These events involve sudden loss of tone or stiffening of the trunk, head, or whole body, and may lead to falls, injury, or slumping [13, 15]. Individual seizure types were analysed separately; therefore, patients may have an increase in one seizure type while experiencing an overall reduction in seizure frequency.

Abbreviation: @6 M, at 6 months follow up.

**TABLE 3:** Seizure outcomes at last follow‐up by seizure type.

	Baseline, *n*	FU, *n*	Increased@FU, *n* (%)	Unchanged@FU, *n* (%)	Improved seizure frequency					Discontinued@FU, *n* (%)	Deceased by last FU, *n* (%)
					0%–30%@FU, *n* (%)	31%–50%@FU, *n* (%)	51%–75%@FU, *n* (%)	76%–90%@FU, *n* (%)	> 90%@FU, *n* (%)		
‘Drop Seizures’ (any)	78	66	0 (0.0)	0 (0.0)	0 (0.0)	18 (23.1)	22 (28.2)	18 (23.1)	8 (10.3)	8 (10.3)	4 (5.1)
Tonic	57	53	1 (1.8)	3 (5.3)	0 (0.0)	16 (28.1)	14 (24.6)	13 (22.8)	6 (10.5)	2 (3.5)	2 (3.5)
Atonic	11	9	0 (0.0)	1 (9.1)	0 (0.0)	1 (9.1)	2 (18.2)	2 (18.2)	3 (27.3)	2 (18.2)	0 (0.0)
GTCS	69	57	1 (1.4)	3 (4.3)	0 (0.0)	17 (24.6)	23 (33.3)	6 (8.7)	7 (10.1)	8 (11.6)	4 (5.8)
Head Drops	4	3	0 (0.0)	0 (0.0)	0 (0.0)	2 (50.0)	1 (25.0)	0 (0.0)	0 (0.0)	1 (25.0)	0 (0.0)
Drop attacks (as labelled by caregivers)	12	12	1 (8.3)	2 (16.7)	0 (0.0)	3 (25.0)	3 (25.0)	3 (25.0)	0 (0.0)	0 (0.0)	0 (0.0)
Absences	39	36	2 (5.1)	7 (17.9)	0 (0.0)	5 (12.8)	10 (25.6)	4 (10.3)	8 (20.5)	3 (7.7)	0 (0.0)
Myoclonic Seizures	23	21	1 (4.3)	2 (8.7)	0 (0.0)	7 (30.4)	7 (30.4)	2 (8.7)	2 (8.7)	1 (4.3)	1 (4.3)
Focal Seizures (with or without impaired awareness)	26	22	4 (15.4)	3 (11.5)	1 (3.8)	4 (15.4)	3 (11.5)	1 (3.8)	6 (23.1)	3 (11.5)	1 (3.8)
Non‐convulsive status	3	3	0 (0.0)	0 (0.0)	0 (0.0)	0 (0.0)	2 (66.7)	0 (0.0)	1 (33.3)	0 (0.0)	0 (0.0)
Other/unspecified	13	13	0 (0.0)	3 (23.1)	0 (0.0)	5 (38.5)	3 (23.1)	0 (0.0)	2 (15.4)	0 (0.0)	0 (0.0)
All Seizure types	79	67	0 (0.0)	0 (0.0)	0 (0.0)	14 (17.7)	27 (34.2)	18 (22.8)	8 (10.1)	8 (10.1)	4 (5.1)

*Note:* Seizure type classification was based on caregiver seizure charts. Throughout the table, *n* refers to the number of patients. Percentages at last follow‐up are calculated using the baseline number of patients for each seizure type. ‘“FU, *n*” includes only patients with available 6‐month data; discontinued or deceased patients are excluded from this column but retained in the baseline denominator for percentage calculations.’ ‘Drop seizures’ (any) includes patients with one or more of the following seizure types: tonic, atonic, tonic–clonic, head drops, or drop attacks (as labelled by caregivers), based on seizure diaries. These events involve sudden loss of tone or stiffening of the trunk, head, or whole body, and may lead to falls, injury, or slumping [13, 15]. Individual seizure types were analysed separately; therefore, patients may have an increase in one seizure type while experiencing an overall reduction in seizure frequency.

Abbreviation: @FU, at last follow‐up.

At 6 months, among those with ‘Drop Seizures’ as defined above (*n* = 78), 50% (39/78) achieved a > 50% reduction in ‘drop seizure’ frequency, including 20.5% with a 51%–75% reduction, 20.5% with a 76%–90% reduction and 9.0% achieving > 90% reduction. A small proportion (6.4%) had < 30% reduction. Across all seizure types at 6 months, 59.5% (47/79) achieved > 50% reduction with 6.3% > 90% reduction. Three patients (3.8%) had < 30% reduction and discontinued treatment shortly after the 6‐month mark due to lack of efficacy. Further analysis by seizure type at 6 months showed that 47.8% (11/23) with myoclonic seizures and 48.7% (19/39) with absence seizures achieved a > 50% reduction in seizure frequency.

At last follow‐up, seizure control remained improved. Among patients with ‘Drop Seizures’ (*n* = 78), 61.5% (48/78) achieved more than 50% reduction: 28.2% with a 51%–75% reduction, 23.1% with a 76%–90% reduction and 10.3% > 90% reduction. Across all seizure types, 67.1% (53/79) achieved more than 50% reduction: 34.2% with a 51%–75% reduction, 22.8% with a 76%–90% reduction and 10.1% > 90% reduction. Further analysis by seizure type at last follow‐up showed that 47.8% (11/23) with myoclonic seizures and 56.4% (22/39) with absence seizures achieved a > 50% reduction in seizure frequency. Reported increases in specific seizure types are listed in Tables 2 and 3 and were few.

Exploratory subgroup analyses were performed to assess whether different aetiologies were associated with cannabidiol response. There were no statistically significant differences in > 50% responder rates between patients with structural and genetic aetiologies at either 6 months (Fisher's exact test, *p* = 0.485) or last follow‐up (Fisher's exact test, *p* = 0.319). Similarly, there were no statistically significant differences between patients with an active VNS and those without an active VNS at either 6 months (Fisher's exact test, *p* = 0.058) or last follow‐up (Fisher's exact test, *p* = 0.364). Only one patient was receiving a ketogenic diet; therefore, subgroup analysis was not performed.

#### 3.3.2 Clobazam Dose and Response

Forty‐one patients (52%) were already on maintenance clobazam prior to cannabidiol initiation, while 37 patients (47%) were started on clobazam in accordance with NICE requirements. One patient was on nitrazepam due to clobazam intolerance. In cases where clobazam was historically poorly tolerated, minimal or small doses were used: ≤ 2.5 mg/day in 29 patients including ≤ 1 mg/day in 14 patients. Table 4 shows ‘drop seizure’ response rates stratified by clobazam dose. Higher clobazam doses showed a numerically greater proportion of responders. However, statistical analysis using chi‐squared did not reveal significant differences between dose groups. No clear dose–response trend was detected using group comparisons.

**TABLE 4:** Drop seizure response by clobazam dose.

Clobazam daily dose groups at last follow‐up	*n*	< 50% Drop Seizure Reduction	> 50% Drop Seizure Reduction	51%–75%	76%–90%	> 90%
≤ 1 mg	14	10	4	3	1	0
> 1 & ≤ 5 mg	27	13	14	6	8	0
> 5 mg	36	15	21	7	7	7

#### 3.3.3 Seizure‐Free Days

The mean number of seizure‐free days per month increased from 5.7 ± 8.8 at baseline to 11.1 ± 9.4 at 6 months (*n* = 75; excluding 4 patients who discontinued treatment), showing a statistically significant improvement (paired *t*‐test, *p* < 0.001). At the last follow‐up, the mean number of seizure‐free days per month further increased to 12.5 ± 10.6 days per month (*n* = 62; 8 patients had discontinued treatment, 4 had died, and data were unavailable for 5 patients), remaining significantly higher than baseline (paired *t*‐test, *p* < 0.001).

#### 3.3.4 Rescue Medication Use

At six‐month follow‐up, rescue medication use had reportedly decreased in 36 of 75 patients and increased in five. By the last follow‐up, a reduction in rescue medication use was reported in 31 of 64 patients and increased in three (excluding the four patients who had died, eight who had discontinued cannabidiol, and three with missing data).

#### 3.3.5 Hospital Admissions Related to Seizures or Seizures Related Injuries

Carers reported a decrease in emergency hospital admissions due to seizures compared with baseline from 0.21 to 0.08 at 6 months in 62 patients where data was available.

### 3.4 Retention

At last follow‐up, 8 patients (10.1%) had discontinued cannabidiol due to adverse events and/or lack of efficacy and 4 patients died of unrelated causes. The most common reasons for treatment discontinuation were sedation/lethargy or somnolence (4/8), behavioural or mood changes (3/8), gastrointestinal intolerance including diarrhoea or vomiting (3/8), and limited efficacy (3/8). Some patients had more than one contributing reason for discontinuation. Only one patient discontinued because of an increase in seizures. Individual reasons for discontinuation are shown in Table S1.

**FIGURE 2:**
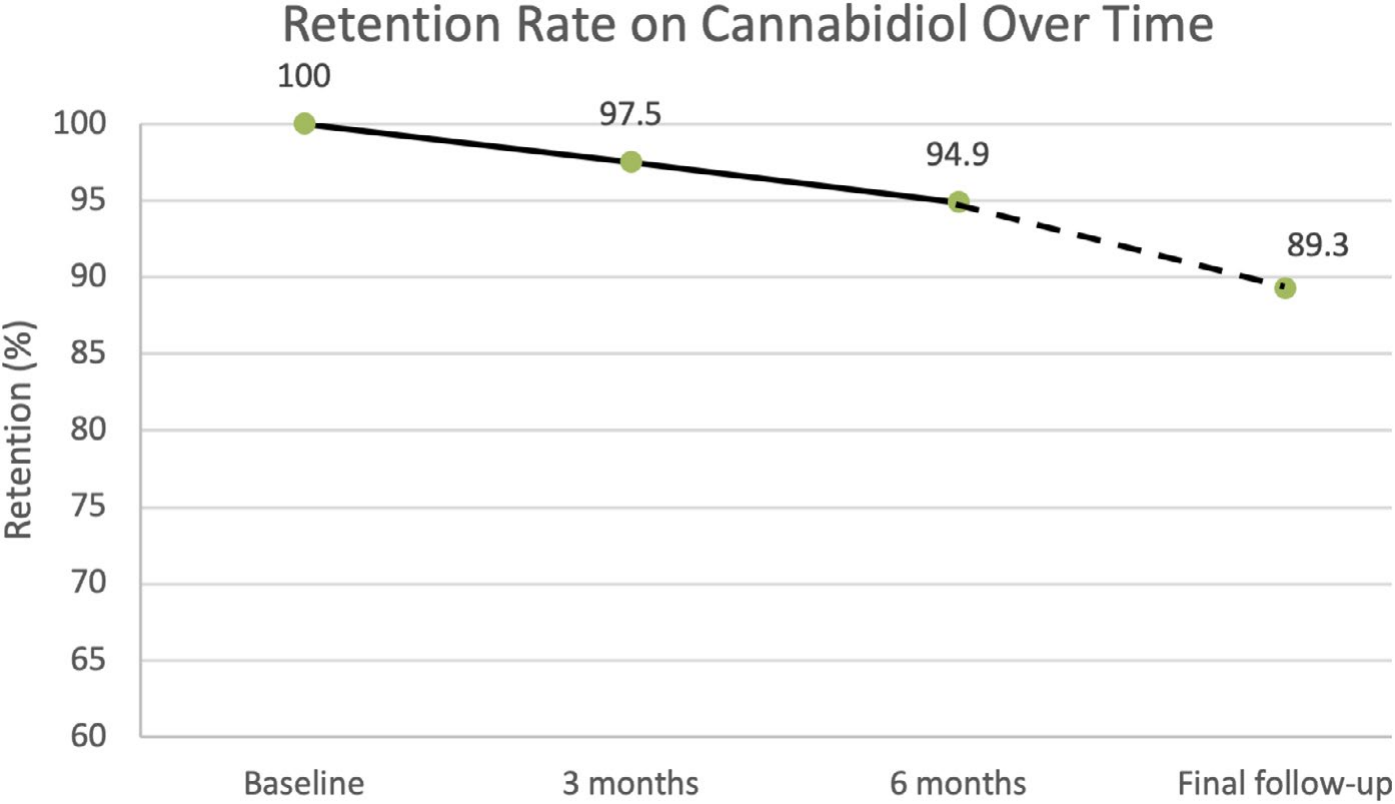
Retention rate on cannabidiol over time (dotted line reflects variable time frame).

Retention was 97.5% at 3 months, 94.9% at 6 months and 89.3% at last follow‐up (Figure 2). Cannabidiol retention did not differ significantly according to concomitant valproate use (38/41 vs. 33/38; Fisher's exact test, *p* = 0.471). None of the discontinuations in the valproate group were related to valproate or liver function abnormalities. Seventeen patients were still undergoing dose titration at the last follow‐up. Similarly, treatment retention did not differ significantly according to aetiology (Fisher's exact test, *p* = 0.719) or active VNS status (Fisher's exact test, *p* = 0.342).

### 3.5 Adverse Events

Reported adverse events are listed in Table 5. The most frequently reported adverse events were drowsiness (33), diarrhoea (24), deranged liver function tests (LFTs) (12) and worsening behaviour (11), often due to drug–drug interaction requiring reductions in concomitant medication described below. LFT abnormalities were more frequent in patients receiving concomitant valproate than in those not receiving valproate (10/39 vs. 2/40; Fisher's exact test, *p* = 0.013). These responded to adjustments in valproate; none of these cases led to treatment withdrawal. In addition, statistical analysis did not show a significant difference in > 50% seizure response between these groups. A decrease in platelet count was observed in two patients and also improved following a reduction in valproate dose. Diarrhoea was reported in 24 patients, with treatment discontinued in two cases. Symptoms often improved with dose reduction. Once symptoms settled, dose titration proceeded more slowly; however, in 17 patients, diarrhoea limited the maximum cannabidiol dose that could be used.

**TABLE 5:** Frequency of adverse events reported during cannabidiol treatment.

Reported Adverse Events	*n*
Drowsiness	33
Diarrhoea	24
Deranged liver function tests	12
Worsening behaviour	11
Increased seizure frequency	6
Gastrointestinal upset (other than diarrhoea)	5
Tiredness	4
Weight loss	4
Reduced appetite	3
Poor balance	3
Ataxia, mood changes, drooling/dribbling, weight gain, restlessness/agitation, low platelet count	2 each
Disorientation, muscle cramps, disturbed sleep, slurred speech, floppy, impaired mobility, poor taste, poor memory, falls, hyponatraemia, lymphopenia, rash	1 each

### 3.6 Medication Adjustments

Clinically significant drug interactions were noted with clobazam, valproate, lamotrigine, everolimus, rufinamide and phenytoin requiring medication adjustments. Interactions were often anticipated and managed with increased monitoring and pre‐emptive guidance and reactive reductions if side‐effects emerged.

Concomitant anti‐seizure medications (ASMs) were withdrawn in 14 patients—1 ASM in 12 patients and 2 ASMs in 2 patients. Dose reductions of other ASMs were made in 51 patients, and 28 of these were able to reduce doses of ≥ 2 ASMs.

**FIGURE 3:**
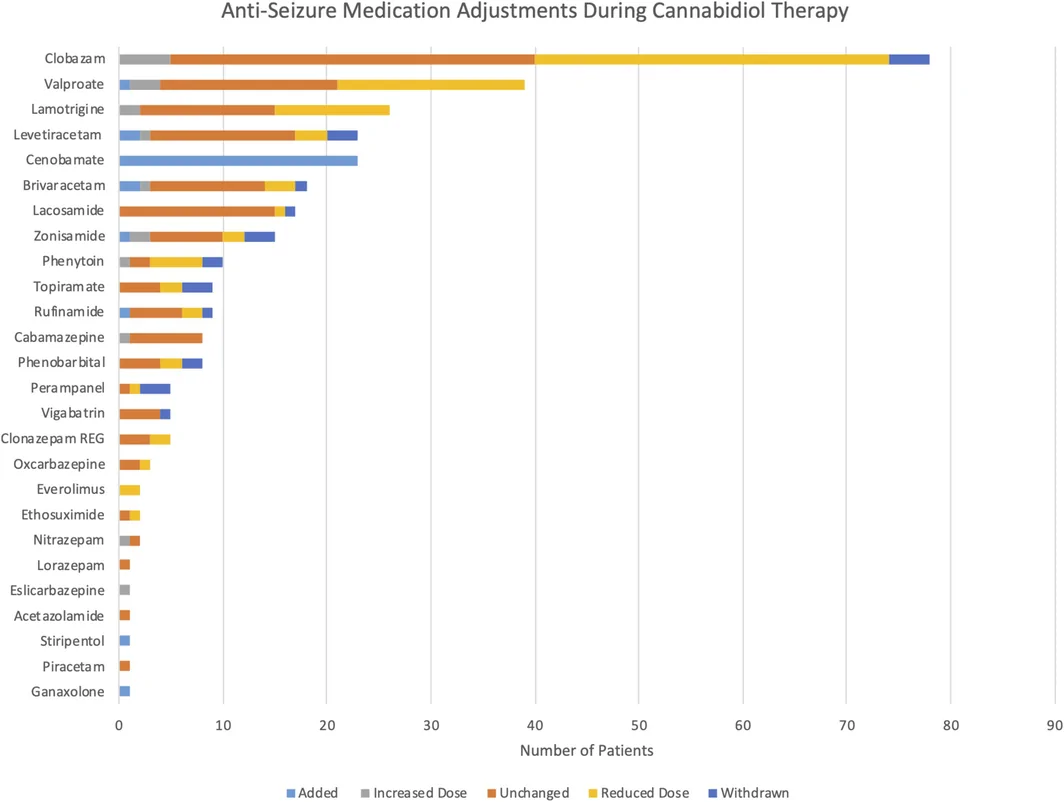
Concomitant anti‐seizure medication adjustments during cannabidiol therapy. All patients received clobazam alongside cannabidiol in accordance with NICE guidance, except one who was on nitrazepam due to clobazam intolerance. Clobazam adjustments shown reflect changes made during cannabidiol therapy. Cenobamate was added in 23 patients. Outcomes reported here relate to outcomes before initiation of any new ASM.

The most commonly withdrawn ASMs were perampanel (*n* = 3) and phenytoin (*n* = 3). Clobazam was reduced in 34 patients, valproate in 18 patients, and lamotrigine in 11 (Figure 3). Dose increases were infrequent but occurred with clobazam (5 patients), valproate (3), lamotrigine (2) and zonisamide (2).

An additional ASM was added in 31 patients, most commonly cenobamate (*n* = 23). Data at last follow‐up reported here relate to outcomes before the addition of another ASM.

Case examples illustrating clinically significant cannabidiol drug interactions are summarised in Table 6.

**TABLE 6:** Case examples illustrating cannabidiol drug interactions.

Concomitant medication	Case examples
Clobazam (high dose)	In a case on high‐dose clobazam, cannabidiol initiation led to significant sedation, dysarthria and cognitive slowing. Clobazam was gradually reduced with subsequent improvement, underscoring the importance of anticipatory clobazam dose adjustment when initiating cannabidiol therapy, particularly in patients on high doses.
Valproate	In a case involving valproate, thrombocytopenia and tremor emerged following cannabidiol initiation. Valproate reduction improved platelet counts and tremor while maintaining good seizure control.
Phenytoin	In a case on phenytoin, cannabidiol led to a significant rise in phenytoin levels, liver enzyme derangement, and ataxia. Dose reduction resolved symptoms, highlighting the need for close monitoring of phenytoin levels and where needed proactive adjustment.
Everolimus	In a case involving everolimus, mouth ulcers recurred after starting cannabidiol which led to dose reduction. Everolimus levels remained elevated on later testing compared to baseline.
Lamotrigine	Lamotrigine dose adjustment was also required in one patient due to behavioural disturbances following cannabidiol initiation, which resolved with lamotrigine dose reduction.

*Note:* The case examples are consistent with the known mechanisms of cannabidiol interactions as described in the SmPC [24]. Clobazam and cannabidiol increase levels of each other's active metabolites, contributing to sedation. Cannabidiol increases exposure to clobazam, phenytoin and everolimus. Concomitant use with valproate is associated with an increased incidence of transaminase elevations. Lamotrigine levels may also rise, although data remain limited [24].

### 3.7 Caregiver Feedback on Cognitive Function Changes

Carer‐reported cognitive improvements were described at last follow‐up in 48/67 (71.6%) (where the information was available). Reported improvement included better attention, communication, engagement and sociability, together with improvements in behaviour or emotional state in some patients. Sleep pattern reportedly improved in 19 patients and behaviour in 15 patients. Some caregivers described improvement in mobility or balance. Although a higher proportion of patients who experienced ≥ 50% seizure reduction had reported cognitive benefits (36/44, 81.8%), compared to those without (16/23, 69.6%), this was not statistically significant (chi‐squared test *p* = 0.404).

## 4 Discussion

Our retrospective review demonstrates that adjunctive cannabidiol therapy is associated with good tolerability and effectiveness in adults with LGS.

At 6 months, 59.5% of patients achieved a ≥ 50% reduction in seizure frequency, increasing to 67.1% at last follow‐up. Improvements were observed across multiple seizure types. In our cohort, 50.0% of patients achieved a ≥ 50% reduction in ‘Drop Seizures’ as defined above at 6 months, increasing to 61.5% at last follow‐up. These real‐world observational findings are consistent with those from pivotal trials mainly focused on paediatric populations. In GWPCARE3 and GWPCARE4, 36%–44% of patients receiving cannabidiol achieved a ≥ 50% reduction in ‘drop seizure’ frequency, compared with 14%–24% in the placebo arms [15, 16, 20]. More recent real‐world studies and two systematic reviews have similarly confirmed the effectiveness across DEEs [22, 25, 26, 27, 28]. Our findings support this and additionally provide long‐term outcomes in an adult LGS population including assessment of treatment retention, seizure‐free days, anti‐seizure medication optimisation and drug–drug interaction management.

Recent real‐world studies have begun to explore whether baseline clinical characteristics influence cannabidiol response in DEEs [26, 27]. Our cohort included a broad range of genetic and structural causes of adult LGS encountered in a tertiary epilepsy service. Our analysis did not show significant associations between structural versus genetic aetiology or active VNS status and either > 50% seizure response or treatment retention. However, these findings should be interpreted cautiously given the modest sample size, the heterogeneity of LGS, and the limited number within individual aetiological subgroups. Larger prospective studies with comprehensive characterisation will be required to determine whether specific genetic or structural aetiologies influence cannabidiol responsiveness.

Beyond seizure control, patients experienced an increase in seizure‐free days, a reduction in rescue medication use and fewer emergency hospital admissions. These outcomes indicate not only reduced seizure burden but also potential improvements in quality of life and reduction in emergency healthcare utilisation. Cognitive and behavioural improvements were reported by caregivers in over 70% of cases on continuing treatment. While subjective and inconsistently captured, these align with emerging anecdotal and observational evidence suggesting broader neuropsychological benefits of cannabidiol in similar patients [29]. In our cohort, no statistically significant association was observed between seizure reduction and reported cognitive improvement.

Treatment was well tolerated overall, with an 89.3% retention rate at last follow‐up. Compared with recent real‐world studies including mixed cohorts of patients with DEEs, our cohort showed higher treatment retention throughout follow‐up. This difference may, in part, reflect differences in study populations, as our cohort consisted only of adults with LGS, while published cohorts included mixed populations with different DEE [22, 27, 28, 30]. Drowsiness, diarrhoea, liver enzyme elevations, and behavioural changes were the most commonly reported adverse effects, managed with dose adjustments. Despite the lower maintenance dose of cannabidiol used in our cohort, effectiveness was comparable to pivotal trials. Diarrhoea was reported in 24 patients, which was more frequent than in clinical trials [15, 16, 20, 21]. In most cases, symptoms improved with dose reduction and slower titration, although this limited the maximum dose used. Liver enzyme derangement occurred more often in patients with concomitant valproate but usually resolved following valproate dose reduction and did not result in cannabidiol discontinuation. Concomitant valproate was not associated with cannabidiol treatment discontinuation in our cohort. In addition, our analysis did not show a significant difference in seizure response between patients receiving concomitant valproate and those not receiving valproate. Decreases in platelet count in two patients improved after valproate dose reduction. Drowsiness or behavioural changes often required reduction in concomitant medication. Significant interactions with clobazam, valproate, lamotrigine, everolimus and phenytoin were observed. These need to be anticipated and managed with pre‐emptive guidance and reactive changes if side‐effects emerge [31, 32, 33]. Clobazam was reduced in 34 patients, valproate in 18 and lamotrigine in 11. Furthermore, ASM rationalisation was achieved: overall 14 patients discontinued at least one ASM, and dose reductions were made in 51 patients (including in ≥ 2 ASMs in 28). Thus, while polypharmacy was common in this patient cohort, cannabidiol facilitated some simplification of ASM regimens. In clinical practise, reduction or withdrawal of concomitant antiseizure medications and rationalisation of polytherapy are important goals. In our cohort, this was observed in some patients. Comparable data remain limited in the published literature [15, 20, 21].

Cannabidiol was offered in accordance with NICE guidance with adjuvant clobazam [13]. The median starting dose of cannabidiol was 2.41 mg/kg twice daily (IQR: 0.46). Lower starting doses were occasionally used in patients considered at higher risk of side effects or pharmacokinetic interactions. Titration frequency varied, with dose increments made at intervals of several weeks based on clinical response, tolerability, and potential drug interactions. Dose adjustment was intentionally more conservative than recommended in the Summary of Product Characteristics, mainly to manage any potential side‐effects emerging with concomitant medication as well as service capacity [24]. Follow‐up intervals and dosing strategies also varied, reflecting real‐world clinical practise.

Where clobazam was not already part of the antiseizure regimen, it was usually introduced at least 2 weeks prior to cannabidiol initiation. In a proportion of patients who had a history of not tolerating standard doses of clobazam before, only small doses were prescribed to satisfy NICE requirements for prescribing cannabidiol. Although a numerically greater reduction in drop seizures was observed in patients receiving > 5 mg/day of clobazam, this was not statistically significant.

Our audit has limitations due to its retrospective nature in a single centre. Seizure frequency data relied on caregiver reporting, which can be associated with recall bias and misclassification of seizure types. However, there was consistent data collection during the audit period and seizure trends were consistent and sufficiently robust to support overall conclusions. Although analyses according to aetiology were performed, the numbers within individual subgroups were small, limiting the power to detect aetiology‐specific differences in treatment response. Cognitive and behavioural improvements were not formally assessed using objective tools and were instead drawn from clinic letters and caregiver feedback in view of the retrospective and real‐world nature of the audit.

In conclusion, although no patients were seizure‐free, our findings support the use of cannabidiol as an effective and generally well‐tolerated adjunctive treatment in adults with LGS. Cannabidiol was associated with a significant reduction in seizure burden, increased seizure‐free days, and reduced need for emergency care, alongside high treatment retention and manageable side effects, which resolved with dose adjustments. Improvements in caregiver‐reported cognitive and behavioural function were also noted. These findings underscore the utility of cannabidiol as an effective and well‐tolerated adjunctive therapy in adult patients with LGS and highlight its potential role in rationalising complex ASM regimens.

## Author Contributions

**Pyae Aung:** investigation, writing – original draft, methodology, validation, writing – review and editing, formal analysis, data curation, resources, project administration. **Debbie Miller:** writing – review and editing, investigation. **Emily Sewell:** investigation, writing – review and editing. **Laura Mantoan Ritter:** investigation, writing – review and editing, validation, methodology. **Evangelia Theochari:** investigation, writing – review and editing. **Ioannis Stavropoulos:** investigation, writing – review and editing, formal analysis, supervision, resources, validation, methodology, writing – original draft. **Robert Delamont:** investigation, writing – review and editing. **Mark P. Richardson:** investigation, writing – review and editing. **Joel S. Winston:** investigation, writing – review and editing, writing – original draft, formal analysis, supervision, data curation, resources, validation, methodology, project administration. **Lina Nashef:** investigation, funding acquisition, writing – original draft, methodology, validation, writing – review and editing, formal analysis, project administration, data curation, supervision, resources.

## Funding

Pyae Aung's research fellowship was funded by Jazz Pharmaceuticals. The funder had no role in the design, data collection, analysis, interpretation, or writing of this work. Jazz Pharmaceuticals reviewed the manuscript and had the opportunity to provide comments for the authors to consider.

## Conflicts of Interest

Pyae Aung: Research fellowship post was funded by Jazz Pharmaceuticals. The funder had no role in the design, data collection, analysis, interpretation, or writing of this work. Jazz Pharmaceuticals reviewed the manuscript and had the opportunity to provide comments for the authors to consider. Lina Nashef: Fees for advisory board participation, lectures, and research support from Jazz Pharmaceuticals, UCB, Angelini Pharma and Livanova. Joel S. Winston: Research support from Jazz Pharmaceuticals, Angelini Pharma, and UKRI (MR/W006251/1). Co‐founder of NeuralPulse Ltd. Laura Mantoan Ritter: No conflicts of interest. Debbie Miller: Fees received from Angelini Pharma for co‐chair role and from Jazz Pharmaceuticals for advisory board participation (2023). Emily Sewell: previously received partial funding for epilepsy post from Angelini pharma. Ioannis Stavropoulos: research support from UCB and honoraria from Jazz Pharmaceuticals. Mark P. Richardson: Ad hoc advisory board member for Lundbeck, UNEEG Medical, Piramidal. Co‐founder of NeuralPulse Ltd. Dr. Evangelia Theochari: honoraria for lectures from Eisai and Jazz Pharmaceuticals. Robert Delamont: No conflicts of interest.

## Supporting information


**Table S1:** Individual reasons for discontinuation of treatment.
**Table S2:** Confirmed genetic diagnoses in the cohort.

## Data Availability

The research data are not publicly available because they contain confidential patient information.
