# Supplementary material for: Cannabidiol in Adults With Lennox–Gastaut Syndrome: Real‐World Experience
Source: Eur J Neurol. 2026 Sep 26;33(10):e70760. doi: 10.1111/ene.70760 (PMC13615429; doi:10.1111/ene.70760)
Supplement: Supplementary file 1 [file ENE-33-e70760-s001.docx]

**Supplementary appendix**

**Supplementary Table 1**

Individual reasons for discontinuation of treatment

| **Patient** | **Reason for discontinuation** |
| --- | --- |
| 1 | Initial seizure improvement, but discontinued because of mood changes, lethargy, unsteadiness and behavioural deterioration despite dose reduction |
| 2 | Behavioural deterioration with limited seizure benefit |
| 3 | Diarrhoea |
| 4 | Rash (day 9) |
| 5 | Diarrhoea, spots on buttock, sleepiness and low mood with limited efficacy |
| 6 | Vomiting and excessive daytime somnolence |
| 7 | Lack of efficacy |
| 8 | Worsening seizure frequency |

**Supplementary Table 2**

Confirmed genetic diagnoses in the cohort

| **Genetic diagnosis** | **Gene/Chromosomal abnormality** | **n** |
| --- | --- | --- |
| WDR62-related disorder | *WDR62* | 1 |
| NEXMIF-related intellectual disability and epilepsy | *NEXMIF* | 2 |
| PPP2R5D-related disorder | *PPP2R5D* | 1 |
| CHD8-related disorder | *CHD8* | 1 |
| HNRNPU-related disorder | *HNRNPU* | 1 |
| PURA-related neurodevelopmental disorder | *PURA* | 1 |
| STXBP1-related disorder | *STXBP1* | 1 |
| NAA10-related disorder | *NAA10* | 1 |
| KBG syndrome | *ANKRD11* | 1 |
| SCN1B-related epilepsy | *SCN1B* | 1 |
| CDKL5 deficiency disorder | *CDKL5* | 1 |
| Tuberous sclerosis complex | *TSC1* (1); not available (1) | 2 |
| GLUT1 deficiency syndrome | *SLC2A1* | 1 |
| Down syndrome | Trisomy 21 | 1 |
| Ring chromosome 20 syndrome | Ring chromosome 20 | 1 |
| 15q11–q13 duplication syndrome | Chromosome 15q11.2–q13.1 duplication | 1 |
| Chromosome 15 tetrasomy | Tetrasomy of chromosome 15 | 1 |
| 22q duplication syndrome | Chromosome 22q duplication | 1 |
| Chromosome 15 anomaly | Historical report unavailable | 1 |
